# Effect of exercise-based cancer rehabilitation via telehealth: a systematic review and meta-analysis

**DOI:** 10.1186/s12885-024-12348-w

**Published:** 2024-05-17

**Authors:** Ladislav Batalik, Katerina Chamradova, Petr Winnige, Filip Dosbaba, Katerina Batalikova, Daniela Vlazna, Andrea Janikova, Garyfallia Pepera, Hammoda Abu-Odah, Jing Jing Su

**Affiliations:** 1 Department of Rehabilitation, University Hospital Brno, Brno, Czech Republic; 2 Department of Public Health, Faculty of Medicine, Masaryk University, Brno, Czech Republic; 3 Department of Physiotherapy and Rehabilitation, Faculty of Medicine, Masaryk University, Brno, Czech Republic; 4 Department of Rehabilitation and Sports Medicine, Second Faculty of Medicine, Motol University Hospital, Charles University, Prague, Czech Republic; 5 Department of Neurology, Center for Neuromuscular Diseases (Associated National Center in the European Reference Network ERN EURO-NMD), University Hospital Brno, Brno, Czech Republic; 6 Department of Internal Medicine-Hematology and Oncology, University Hospital Brno, Brno, Czech Republic; 7 Clinical Exercise Physiology and Rehabilitation Research Laboratory Department of Physiotherapy, Faculty of Health Sciences, University of Thessaly, Lamia, Greece; 8 School of Nursing, The Hong Kong Polytechnic University, Hong Kong SAR, China; 9 School of Nursing, Tung Wah College, Hong Kong, China

**Keywords:** Exercise-based rehabilitation, Telehealth, Cancer rehabilitation, Home-based exercise, Cancer rehabilitation

## Abstract

**Purpose:**

Exercise-based cancer rehabilitation via digital technologies can provide a promising alternative to centre-based exercise training, but data for cancer patients and survivors are limited. We conducted a meta-analysis examining the effect of telehealth exercise-based cancer rehabilitation in cancer survivors on cardiorespiratory fitness, physical activity, muscle strength, health-related quality of life, and self-reported symptoms.

**Methods:**

PubMed, Web of Science, and reference lists of articles related to the aim were searched up to March 2023. Randomized controlled clinical trials were included comparing the effect of telehealth exercise-based cancer rehabilitation with guideline-based usual care in adult cancer survivors. The primary result was cardiorespiratory fitness expressed by peak oxygen consumption.

**Results:**

A total of 1510 participants were identified, and ten randomized controlled trials (*n* = 855) were included in the meta-analysis. The study sample was 85% female, and the mean age was 52.7 years. Meta-analysis indicated that telehealth exercise-based cancer rehabilitation significantly improved cardiorespiratory fitness (SMD = 0.34, 95% CI 0.20, 0.49, I2 = 42%, *p* < 0.001) and physical activity (SMD = 0.34, 95% CI, 0.17, 0.51, I2 = 71%, *p* < 0.001). It was uncertain whether telehealth exercise-based cancer rehabilitation, compared with guideline-based usual care, improved the quality of life (SMD = 0.23, 95%CI, -0.07, 0.52, I2 = 67%, *p* = 0.14) body mass index (MD = 0.46, 95% CI, -1.19, 2.12, I2 = 60%, *p* = 0.58) and muscle strength (SMD = 0.07, 95% CI, -0.14, 0.28, I2 = 37%, *p* = 0.51).

**Conclusion:**

This meta-analysis showed that telehealth exercise cancer rehabilitation could significantly increase cardiorespiratory fitness and physical activity levels and decrease fatigue. It is uncertain whether these interventions improve quality of life and muscle strength. High-quality and robust studies are needed to investigate specific home-based exercise regimens in different cancer subgroups to increase the certainty of the evidence.

**Supplementary Information:**

The online version contains supplementary material available at 10.1186/s12885-024-12348-w.

## Introduction

According to the latest global cancer data estimates cancer burden rose by approximately 19 million new cases and ten million cancer deaths in 2020. Cancer incidence is expected to continue to rise, with the global cancer burden projected to be 50% higher in 2040 than in 2020 [1, 2]. Therefore, it is essential to develop sustainable approaches to cancer treatment and prevention. While improving cancer treatment and supportive care has reduced mortality rates, many individuals still experience continuing physical and psychological cancer treatment-related side effects, especially fatigue, pain, muscle loss, or depression [3–6]. In addition, adverse effects on the cardiovascular system and worsened cardiovascular risk of survivors have recently been demonstrated, supporting the development of the field of cancer [7, 8]. Therefore, there is a need for long-term systematic supportive care for cancer, highlighting the need for evidence-based rehabilitation interventions tailored to this population.

Exercise-based interventions are increasingly recognized as a cornerstone of rehabilitation for cancer patients and survivors [9]. Evidence from meta-analyses demonstrates that exercise and physical activity can provide a range of physical and psychosocial benefits that can reduce the side effects of cancer treatment [10, 11]. The latest research has demonstrated that exercise-based rehabilitation can significantly enhance cancer survivors' quality of life, cardiorespiratory fitness, and fatigue [12]. Despite the benefits, geographic barriers, a deficit of rehabilitation centers, low referrals, and other factors limit access to exercise-based rehabilitation programs [13].

Telehealth has the potential to revolutionize healthcare delivery by enhancing accessibility, reducing costs, improving quality, and personalizing medicine for patients [14]. In recent years, telehealth has gained popularity as a viable solution to the challenges faced by cancer survivors seeking access to exercise-based rehabilitation programs. An increasing number of smartphone users and internet coverage have made telehealth an attractive approach to the challenges of a resource-limited healthcare system [15]. The integration of telehealth faces several challenges, including regulatory issues, security concerns, and a need for more scientific recommendations [16]. Despite these challenges, telehealth has been shown to be a feasible and effective alternative in different healthcare fields, including cancer rehabilitation [17]. Furthermore, telehealth has enabled access to rehabilitation delivery during the pandemic, providing increased safety and convenience for a burdened patient population and holding the potential to elevate beyond the current best practice [18].

This topic delves into the effectiveness of exercise-based cancer rehabilitation via telehealth and its potential to improve outcomes for cancer survivors. The aim is to provide insights into the feasibility and efficacy of telehealth-based rehabilitation interventions and analyse their impact on cancer survivors' physical and psychological outcomes.

## Methods

A comprehensive literature search was carried out to determine the impact of telehealth-based exercise interventions on cancer patients and survivors. The systematic review was conducted following the Preferred Reporting Items for Systematic Reviews and Meta-Analyses (PRISMA) Guidelines 2020 [19], and the review protocol was registered in the Prospective Register of Systematic Reviews (PROSPERO) registry (CRD42023395521).

### Eligibility criteria

The Populations, Interventions, Comparisons, Outcomes, and Study Designs (PICOS) framework was used to describe eligibility criteria. The inclusion criteria were: 1) P – patients or survivors with a medical diagnosis of cancer during or post treatment (e.g., chemotherapy, surgery); 2) I – intervention arm received telehealth exercise-based cancer rehabilitation intervention delivered by Information and communication technologies (e.g., smartphone, web-platform, internet, or video-monitoring) including the use of telemonitoring and telecoaching tools (e.g., telephone calls, text messages, emails); 3) C – control comparator group received conventional treatment/ rehabilitation, usual care, or waitlist intervention. 4) O – outcomes reported were cardiorespiratory fitness, physical activity levels, quality of life, fatigue, pain, muscle strength, body mass index and occurrence of adverse events; and 5) S – randomized controlled trials (RCTs). The searches were limited to studies published in English. The description of the exercise intervention consisted of identifying the following components: telemonitoring of the exercise and the telecoaching method. Further, a description of the exercise was included: prescription of intensity and session duration using wearable sensors (e.g., heart rate monitors, accelerometers, or pedometers). Description of telecoaching/teleconsulting: the variability of supervising, educating, and motivating approach for physical exercise.

The criteria for exclusion were defined as 1) quasi-experimental, qualitative, or case studies; 2) study protocols; 3) conference abstracts; and 4) full-text articles that were unavailable even after contacting the authors.

### Search methods

An electronic literature search was conducted in March 2023 through the PubMed database and the Web of Science metasearch engine. The inclusion of these two databases is to allow a comprehensive search as PubMed focuses mainly on medicine and biomedical sciences with large number of keywords per search, whereas Web of Science cover most scientific fields. Web of Science includes the oldest publications with archived records dated back to 1900 [20]. The search was structured to identify the effect of telehealth exercise interventions published since 2000 in English. Search terms included exercise-based terminology, cancer, and telehealth medicine terms. Telehealth, in exercise-based terminology, involves the use of technology to deliver remote healthcare services, including virtual consultations, exercise prescriptions, and monitoring. The detailed selection process involved a keyword search summarized in Supplementary Table S1. Authors conducting the study selection process hand-searched the references of topical systematic reviews to identify relevant studies not captured in the search.

The relevant articles were chosen based on keywords after an initial literature search. Two independent reviewers (KF & PW) manually assessed the articles based on their titles and abstract. After the first round, the full text of the articles was assessed for relevance based on the inclusion criteria. Any disagreements were resolved by consensus or by discussing with a third reviewer (KB). All reports were combined in cases with multiple publications for a study, and the version with the most systematic data was selected for analysis. The authors of the studies were also contacted to request additional information when necessary.

### Study quality and risk of bias

Two researchers (JJS & LB) assessed methodological quality independently using the Cochrane risk of bias tool 2.0. Study quality was assessed concerning seven domains of bias: allocation bias, selection bias, performance bias, attrition bias, detection bias, reporting bias, and an auxiliary domain (other bias) [21]. The bias domains of the tool were identified to purposefully cover all fundamental bias mechanisms in RCTs and it is the most commonly used tool (about 100% for Cochrane reviews and 31% for non-Cochrane reviews) [21]. Risk of bias was judged as unclear or high when data were insufficient or uncertain. The funnel plots analysis was conducted to evaluate publication bias.

### Data extraction

All study data were extracted independently by two authors (KCH & PW), and any disagreement was resolved by discussing with a third author (KB). The authors extracted data, including (**a**) origin of the articles: authors, year, and country; (**b**) sample characteristics: sample size, age, sex, treatment, and diagnosis; (**c**): group design: intervention given to experimental and control group, (**d**) duration, (**e**) health outcomes and instruments, and (**f**) completion rate, (**g**) Frequency, Intensity, Type, and Time within the exercise group.

### Data analysis

Review Manager 5.3 (Nordic Cochrane Centre, Cochrane Collaboration) was used for data pooling when three or more studies reported the same outcome. Cardiorespiratory fitness was the primary outcome. Data from immediately after intervention completion was used for studies that reported outcomes at different endpoints. An intervention effect was expressed as Cohen’s d when studies used different tools/questionnaires to assess the same outcome, calculating standard mean difference (SMD) with a 95% confidence interval (CI) of post-intervention results between groups. Mean difference (MD) was used for pooling studies using the same instrument/tool. Cohen's d > 0.8 represents a large, 0.5–0.8 a medium, and 0.2–0.5 a small effect [22]. Risk ratios were calculated for dichotomous outcomes with the Mantel–Haenszel method. Heterogeneity was evaluated using I^2^ and τ2; I^2^ > 50% and τ2 with *p*‐value < 0.1 suggested significant heterogeneity, and thus, a random effect model was used. Leave-one-out sensitivity analysis was conducted when the pooled effect showed significant heterogeneity (I^2^ > 50%) [23]. Subgroup analysis was performed further to investigate the intervention effect across different study characteristics (e.g., study duration, control group intervention) for outcome variables with at least three studies in each subgroup. In this study subgroup analysis was only conducted for the cardiorespiratory fitness outcome regarding different intervention duration for the limited number of studies available for other outcome parameters.

## Results

**Fig. 1:**
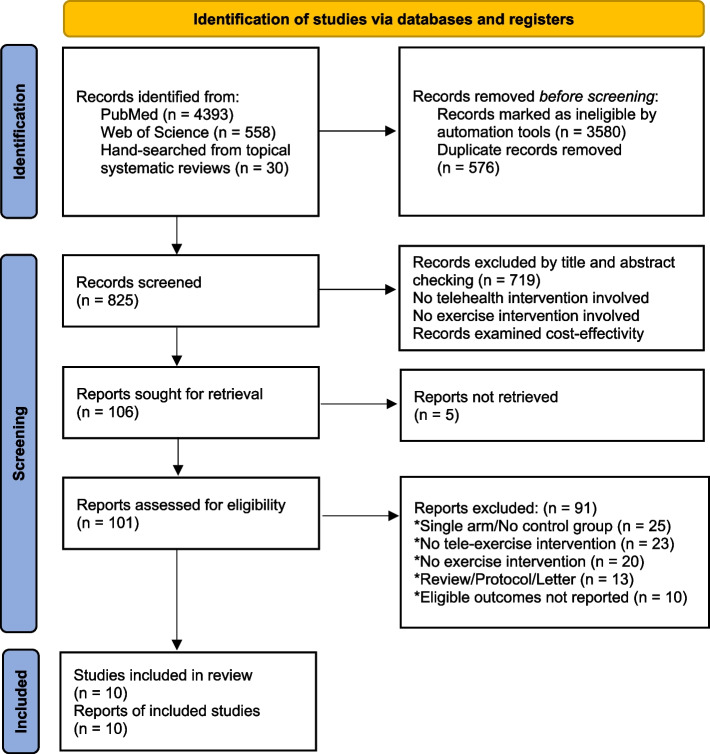
Flow diagram detailing the search strategy

A database and meta-search engine search were performed and identified 4981 records. After screening the titles and abstracts, it was found that 719 publications did not meet the inclusion criteria. Of the 101 full-text publications, 91 records were excluded. Finally, ten publications met the inclusion criteria for this systematic review and meta-analysis. Figure 1 provides an overview of the study flow process.

### Studies included

The characteristics and findings of the ten included studies [24–33] are shown in Table 1. All were of RCT design. The methodological characteristics of studies and adherence and safety outcomes within the studies are presented in Supplementary Table S2 and S3. Three of the ten studies were conducted in the USA [30, 32, 33]; one each was conducted in the United Kingdom [29], Canada [31], France [24], Spain [27], Netherlands [28], Germany [26], and China [25].

**Table 1:** Characteristics and results of studies that evaluated the effects of home-based exercise

Study	Treatment	Cancer	Study	N	Sex	Mean Age	Duration (week)	Exercise program	Intensity Aerobic/Resistance	Exercise reference prescription	Frequency	Monitoring/feedback	Outcomes Primary/ Secondary
Cornette (2016) [24]	During ADT	BC	RCT (1:1)	44	W	51	27	Aerobic exercise, 30–50 min/session + resistance exercise;	1-VT/NS	NS	3/week	HR monitor; exercise diary; phone call 1/week	CRFtns /PA, fatigue, HRQOL, strength
Dong (2019) [25]	Post CT, RT	BC	RCT (1:1)	60	W	50	12	Aerobic exercise, 30 min/session + resistance exercise	RPE 13–16 / 70–80% 1RM	ACSM Guidelines (2018)	4/week	face-to-face televideo 4/week; phone step-recording app;	HRQOL/ CRFtns, muscle strength,
Falz (2023) [26]	Post-surgery	BC/CRC/PC	Multicenter RCT (1:1)	122	M/W	55	12	Strength-endurance video training, 30 min/session	Individual	US Physical activity guidelines (2018)	3/week	Wearable HR monitor, Telemonitoring application	CRFtns /PA, HRQOL, rate-pressure-product, anthropometry,
Galiano-Castillo (2016) [27]	Post CT, RT	BC	RCT (1:1)	81	W	48	8	Aerobic exercise, 90 min/session	60–80 HRmax 11–13 RPE	ACSM Roundtable (2010)	3/week	Online system, video conference sessions 3/week, text message feedback	CRFtns /HRQOL, fatigue, pain, adherence, strength
Gehring (2018) [28]	Post CT, RT or surgery	Gliom	Pilot feasibility RCT (2:1)	34	M, W	48	24	Aerobic exercise	60–85% HRmax	ACSM Guidelines (2014)	3/week	HR monitor; training log online; e-mail 1/week	Accrual, attrition, Adherence/CRFtns PA, body composition – anthropometry, satisfaction
Lahart (2018) [29]	Post ADT or surgery	BC	RCT (1:1)	80	W	52	24	Aerobic exercise; 30 min/session	Moderate	Bull (2010)	Gradually 3–7/week	PA diary recommended; phone call/4 weeks	CRFtns /PA, body composition—anthropometry
Ligibel (2012 ) [30]	Post-surgery, CT, RT	BC/CRC	Multicenter RCT (1:1)	121	M/W	54	16	Aerobic exercise, 180 min/week	Moderate	Holmes (2005)	NS	Telephone-based intervention, 1 call/week	CRFtns /PA, HRQOL, fatigue, anthropometry
McNeil (2019) [31]	Post CT, RT or surgery	BC	RCT (1:1:1)	45	W	58	12	Aerobic exercise; 150–300 min/week,	40–59% HRR / 60–80% HRR	ACSM Roundtable (2010)	NS	HR monitor; exercise diary; phone call or e-mail 1/3 weeks	PA/ CRFtns, body composition – anthropometry
Pinto (2013) [32]	Post-surgery, CT, or RT	CRC	RCT (1:1)	46	M, W	57	12	Aerobic exercise gradually to 30 min/session	64–76% HRmax	ACSM Guidelines (2005)	Gradually 2–5/week	Accelerometer; home PA log; 1 phone call/week	PA/ CRFtns, fatigue, HRQOL
Rogers (2023) [33]	Post treatment	BC	Multicenter RCT (1:1)	222	W	54	12	Aerobic exercise, 40–60% HRR 30–50 min, home-based exercise < 150 min/week	Moderate	Campbell (2019)	3/week	Hybrid; 4 weeks supervised + 8 weeks home-based, counseling / 2 week, HR monitor, exercise diary	CRFtns /PA, strength, fatigue, anthropometry

*1RM* One repetition maximum, *ACSM* American College of Sports Medicine, *CT* Chemotherapy, *RT* Radiotherapy, *RCT* Randomized controlled trial, *HRR* Heart rate reserve, *HR* Heart rate, V*O*_*2*_*max* Maximal oxygen consumption, *ADT* Androgen deprivation therapy, *RPE* Rating of Perceived Exertion, *HRQOL* Health-related quality of life, *HRmax* Maximum heart rate, *BMI* Body mass index, *PA* Physical activity, *PSA* Prostate-specific antigen, *VT* Ventilatory threshold, *CRFtns* Cardiorespiratory fitness, *NS* Not specified, *M* Men, *W* Women, *N* Number of participants, *BC* Breast cancer, *PC* Prostate cancer, *CRC* Colorectal cancer

### Risk of bias assessment

**Fig. 2:**
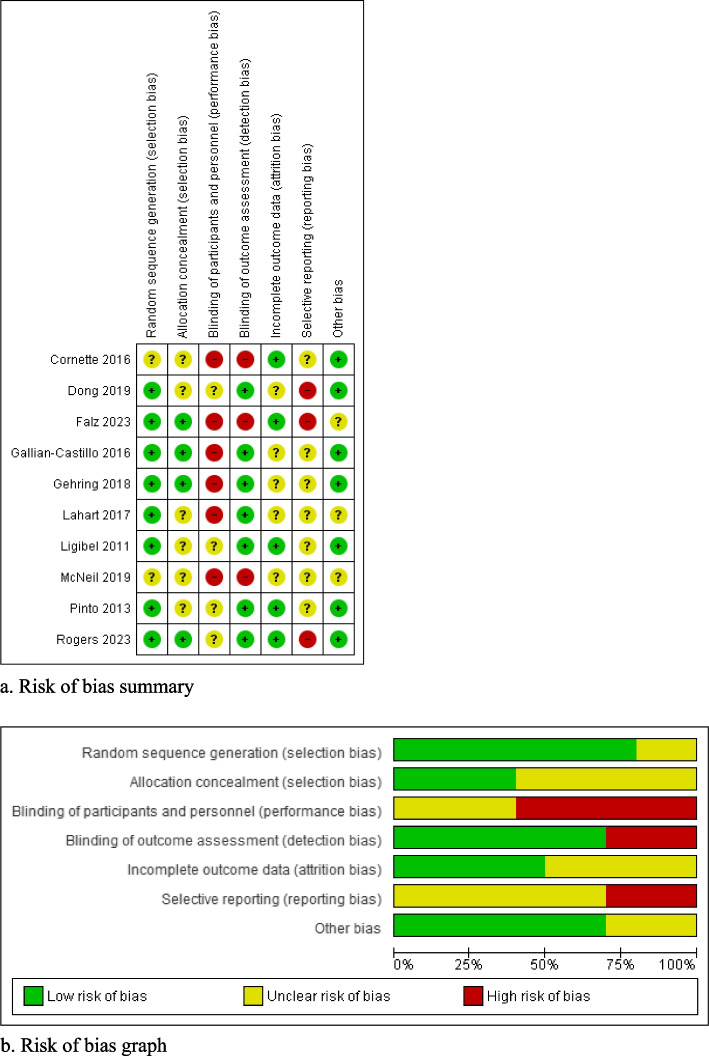
**a** Risk of bias summary. **b** Risk of bias graph

Figure 2a,b summarizes the risks of bias assessment. Two major concerns were the inadequate report of randomization or allocation processes and the selective reporting bias. Referring to trial registration and/or study protocol, three studies were at risk of selective reporting for omitting some proposed outcomes [25, 26, 33]; and the rest provided no registration records for evaluation. Participants were not blinded across the studies, which is understood as a common challenge for eHealth interventions to ensure informed consent. In addition, between-group comparability at baseline was unclear in three studies [26, 29, 31].

### Sampling and recruitment

Sample size calculation was reported in 9/10 studies. The number of participants in the included studies ranged from 34 to 222 participants. A total of 855 participants, with 84.9% representation of the female population, were included in the systematic review. A few studies [28, 32] reported recruitment difficulties and needed to include the target number of participants. Reasons were given as insufficient patient motivation or competing demands on time. Recruitment strategies were reported via email [31]; hospital database and/or hospital referral [28]; a combination of methods, personal recruitment, media and community presentation [32]; postoperatively before discharge [26]; oncologist discussion [27, 30]; specialist nurses [29] or community and clinic-based recruitment [33]. In 2/10 studies, the recruitment method was not specified.

### Characteristics of participants

The participants in the studies were cancer survivors of various types. Table 1 reports information on treatments. Breast cancer was the most common 6/10. Other studies included patients with glioma, colorectal cancer, or combined studies of participants with colorectal/breast cancer or colorectal/breast/prostate cancer. The age of the patients ranged from 37 to 73, with an average of 52.7 years. A wide range of age categories was seen most often.

### Intervention

Telehealth cancer exercise interventions were different in the included studies. The intervention period ranged from 8 to 27 weeks. The studies used various forms of telemonitoring of exercises and telecoaching (consultation, feedback). In most 8/10 studies, the exercise intervention was carried out completely autonomously with post exercise telecoaching [24, 26, 28–33]. The supervised real-time telemonitoring of the exercise was included in 2/10 interventions [25, 27]. Usually, exercise protocols use telemonitoring and post-exercise telecoaching methodology. Half of the studies used HR monitors for telemonitoring exercise intensity [24, 25, 28, 31, 33]. Telecoaching was most often carried out in a telephone call, text messages, or a combination. Telemonitoring and telecoaching in the studies were performed by physiotherapists [26, 28], exercise physiologists [24, 31], exercise specialists [25, 33], or research staff [27, 29, 32]. Telecoaching was provided in the range of 1—4 times a week, most often once weekly. The content of telecoaching included checking compliance with the exercise prescription, feedback, the occurrence of adverse events, technical support, or solving obstacles to exercise.

The included studies varied exercise prescriptions (Frequency, Intensity, Time, and Type). The frequency most often prescribed was 3 exercise sessions per week [24, 26–29, 33]. The total range was 2—5 sessions per week. The methodology for prescribing exercise intensity was determined differently, usually at a moderate intensity level [24, 25, 27–33]. Furthermore, the maximum heart rate obtained by exercise test [27, 28, 30, 31], anaerobic threshold, [24] or evaluation of the rate of perceived exertion on the Borg scale (6—20) [25, 27] was used to reach moderate intensity level. The duration of the exercise session was most often in the range of 30—50 min [24–27, 29, 32]. There were 3/10 studies that prescribed a target time exercise threshold per week [30, 31, 33]. The prescription of the intensity or duration of the exercise session was progressive and individualized in 5/10 studies [24, 26, 27, 32, 33].

There were 3/10 studies that included a combination of aerobic strength training where the aerobic component dominated [24, 30, 32]. The rest of the studies were designed purely aerobically on the principle of exercise-based rehabilitation. Three studies prescribed the exercise modality of walking, cycling, or home exercise on ergometers [24, 30, 32]. Two studies included a range of variable aerobic methods, including walking, cycling, ball games, and swimming [28, 31]. The exercise modality was not adequately defined in four studies in the methodological description [25, 30, 31, 33]. Studies involving resistance training components used bodyweight exercises [26, 27] or resistance bands [24]. Resistance prescription with bodyweight exercises was prescribed two to three times each week and consisted of two sets of 8–15 repetitions for major muscle groups at moderate to vigorous intensity [26, 27]. Exercise using resistance bands was prescribed once weekly and consisted of two sets of 8–12 repetitions on five muscle groups (abdominal, hamstring, quadriceps, triceps, and gluteus maximus) [24]. In half of the studies, warm-up and cool-down phases were included in exercise prescriptions [24–27, 32].

### Control groups

Instructions for control group participants were provided in all studies. Participants from six included studies [24, 26–30] received a physical activity recommendation based on guidelines. Two studies recommended participants to maintain their regular PA [31, 32] and the other two involved supervised exercise in a center [25, 33]. In four studies [28–30, 32], the participants in the control groups were offered an exercise intervention after the end of the study.

### Completion rate

Training diaries [24, 30, 32] or web platform training logs [26–28, 31] assessed adherence to the exercise prescription. Adherence to the exercise protocol was reported relatively consistently (Supplementary Table S2), with only 2/10 studies not reporting adherence to the exercise protocol [25, 29]. A high retention rate (range 74 – 94%) was reported in five studies [24, 26–29, 33]. One study reported high levels of adherence but did not define their methodology [31]. Half of the studies report moderate to high adherence (65—90%) with target exercise intensity [24, 26, 28, 30, 32]. Another study reports high adherence with target exercise time at the correct intensity per week [31]. The remaining 4/10 studies did not report exercise adherence outcomes.

### Occurrence of adverse effects

The occurrence of adverse effects was not reported in half of the studies. Three out of five studies reported no adverse events or death recorded [24, 26, 27]. Gering et al. reported one mild adverse effect associated with exercise, specifically an aggravation of pre-existing osteoarthritis-related knee pain [28], and Rogers et al. reported one serious adverse event associated with exercise, specifically a bone fracture. Another 12 unrelated adverse severe events that occurred were reported [33].

### Outcomes

#### Cardiorespiratory fitness

**Fig. 3:**
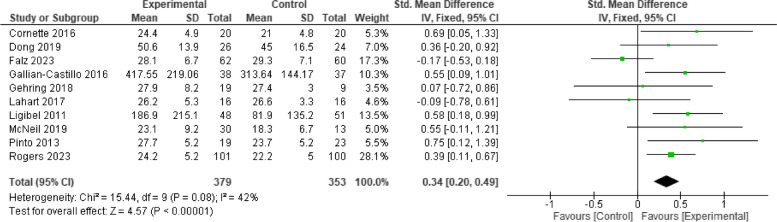
Effect of telehealth exercise on cardiorespiratory fitness

**Fig. 4:**
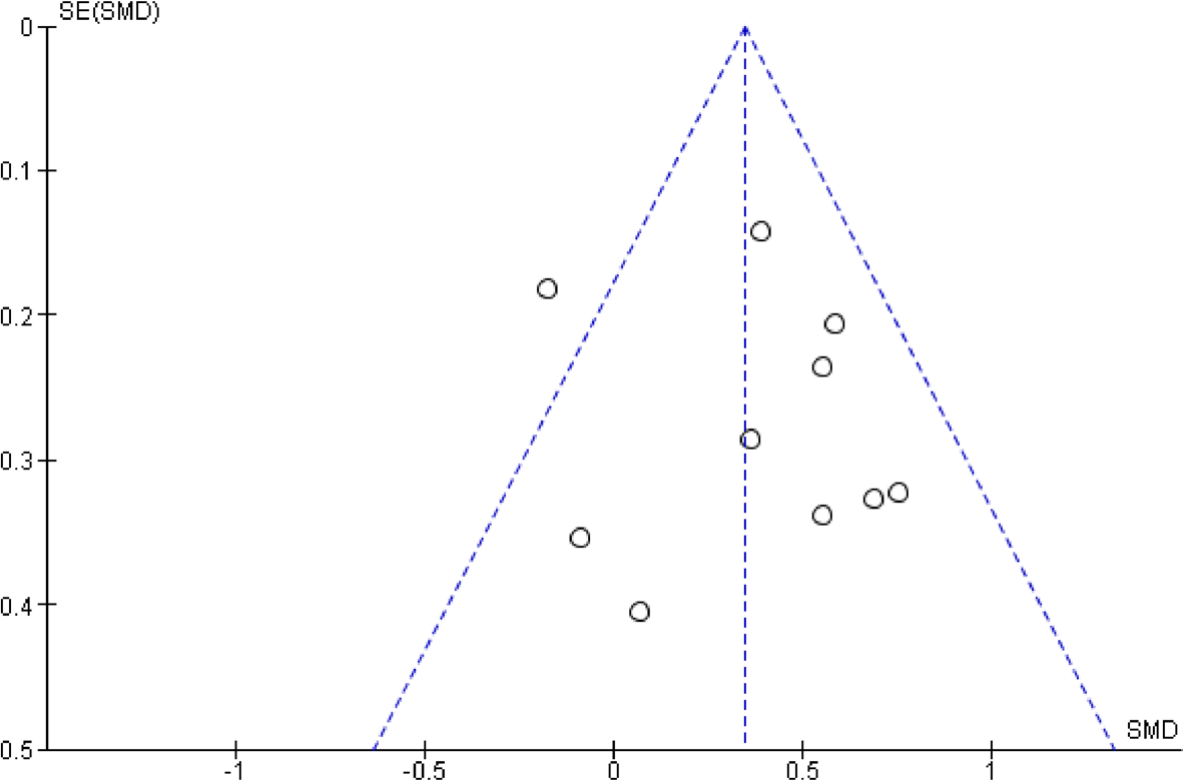
Funnel plot of comparison: cardiorespiratory fitness

All included studies investigated the effect of telehealth exercise-based rehabilitation intervention on cardiorespiratory fitness levels measured by pVO2 and a six-minute walk test. Data pooling of these studies indicated that exercise significantly improved cardiorespiratory fitness [*n* = 10, SMD = 0.34, 95% CI 0.20, 0.49, I2 = 42%, *p* < 0.001] (Fig. 3). Subgroup analysis showed that studies with intervention duration of more than 12 weeks showed a slightly higher magnitude of effect [*n* = 4, SMD = 0.39, 95% CI (0.04, 0.74), I^2^ = 27%, *p* = 0.006] compared with intervention duration ≤ 12wks [*n* = 6, SMD = 0.36, 95% CI (0.08, 0.63), I^2^ = 54%, *p* < 0.001]; both with small effect size. The selection of a 12-week timeframe is based on the frequent use of this duration in early cancer rehabilitation interventions, which allows for a significant improvement in cardiorespiratory fitness level during/immediately after cancer treatment. The funnel plot analysis of all the included studies observed a symmetrical and pyramid-like scatter of points at both sides of the weighted average standard mean difference, indicating low publication bias (Fig. 4). The subgroup analysis figures are presented in Supplementary Fig. 1.

### Physical activity

**Fig. 5:**
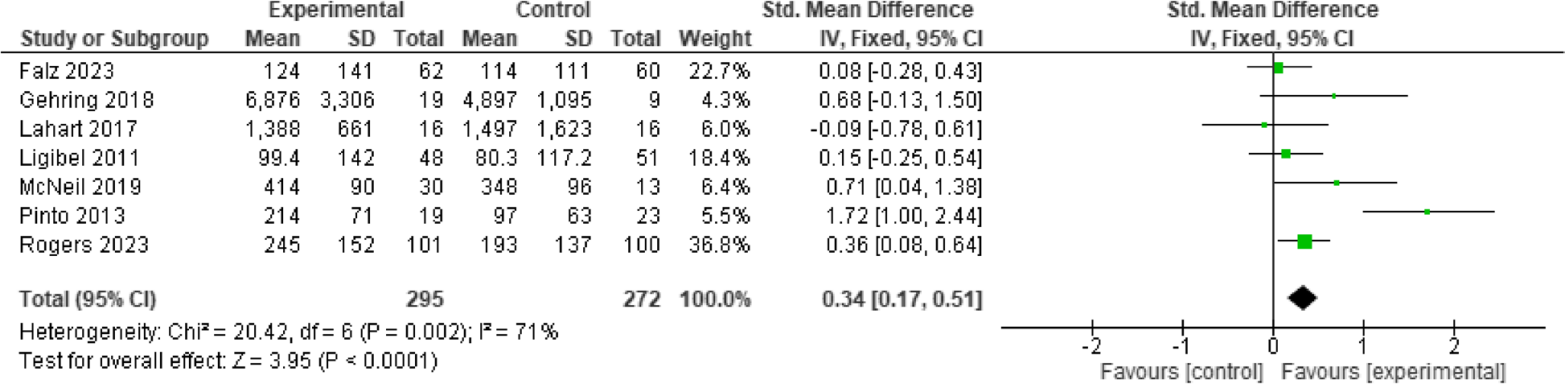
Effect of telehealth cancer exercise on physical activity

Seven studies measured physical activity level in terms of minutes of activities per week, and the intervention showed significant improvement [*n* = 7; SMD = 0.34, 95% CI (0.17, 0.51), I^2^ = 71%, *p* < 0.001]. The high heterogeneity was resolved by removing one study with a large effect size [32] that provided weekly phone calls and an accelerometer to promote daily physical activity; and the significant improvement retained [*n* = 6, SMD = 0.26, 95%CI (0.09, 0.43), *P* = 0.003] (Fig. 5). By removing one that provided control group supervised exercise training, the significant effect of tele-health exercise-based rehabilitation and the heterogeneity remained [*n* = 6, SMD = 0.33, 95% CI 0.12, 0.54, I^2^ = 75%, *p* = 0.002].

### Health-related quality of life

**Fig. 6:**
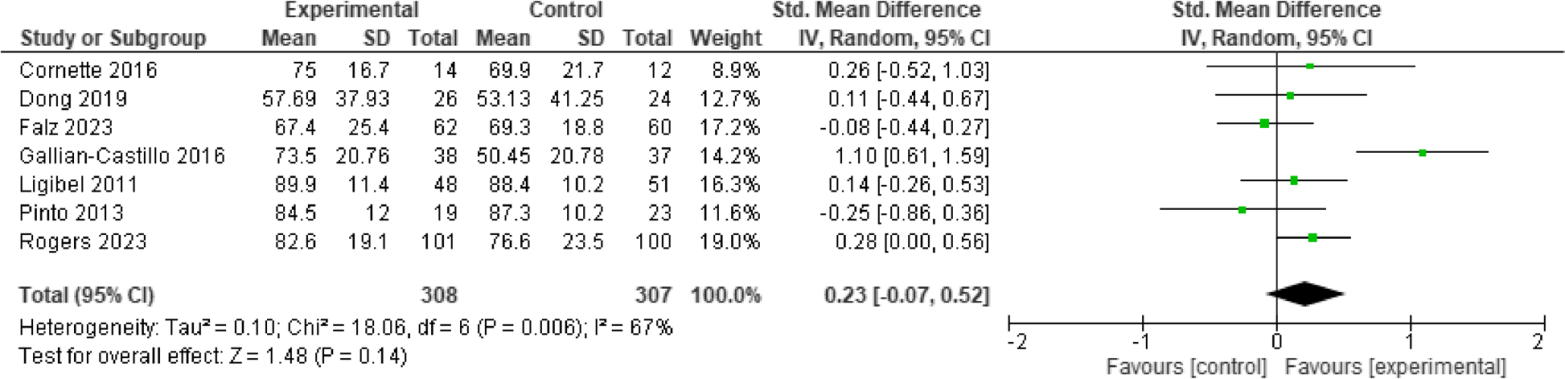
Effect of telehealth cancer exercise on HRQoL

Seven studies measured health-related quality of life using EORTC QLQ C30 (Global status subscale [24, 26, 27] and Physical functioning subscale [30] and SF-36 (Physical functioning subscale) [25, 32, 33]. Data pooling showed no significant improvement of telehealth cancer exercise intervention on HRQoL [*n* = 7; SMD = 0.23, 95%CI (-0.07, 0.52), I^2^ = 67%, *p* = 0.14] (Fig. 6). The sensitivity analysis did not show significant deviation.

### Fatigue

**Fig. 7:**
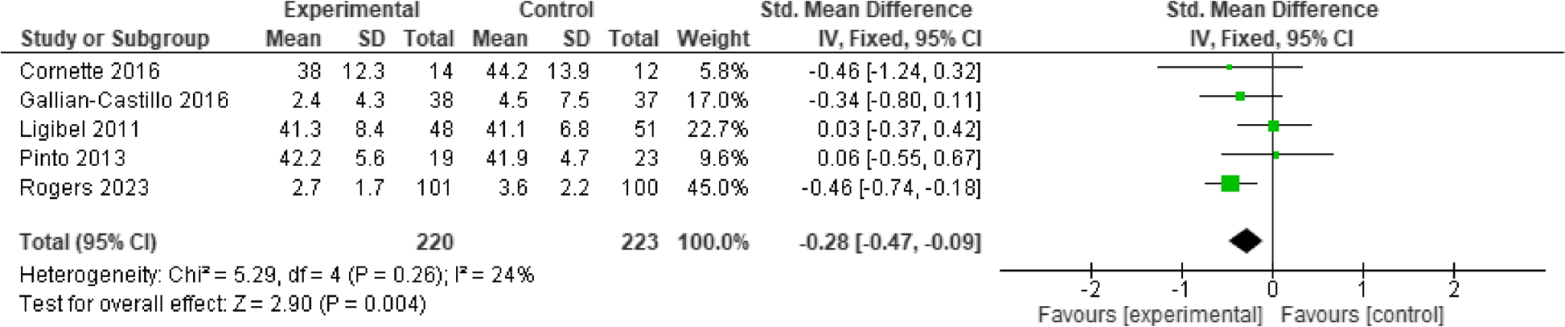
Effect of telehealth cancer exercise on fatigue

Self-reported fatigue was measured by five studies, and data pooling showed significant improvement for patients receiving telehealth exercise [*n* = 5, SMD = -0.28, 95% CI (-0.47, -0.09), I^2^ = 24%, *p* = 0.004] (Fig. 7).

### Body mass index

**Fig. 8:**
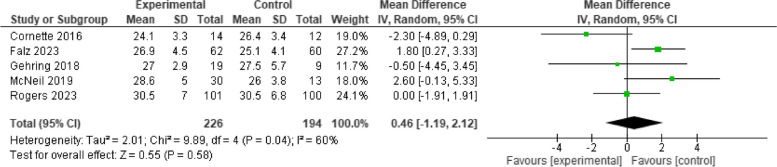
Effect of telehealth cancer exercise on BMI

Body mass index was measured by five studies, and data pooling showed no significant improvement [*n* = 5, MD = 0.46, 95% CI (-1.19, 2.12), I^2^ = 60%, *p* = 0.58]. (Fig. 8).

### Strength

Strength was measured by four studies, including handgrip strength and leg muscle strength [24, 25, 27, 33]. Two studies showed significant improvements [25, 27]. The detailed descriptive results are shown in Table 2.

**Table 2:** Descriptive results of the strength, depression, and anxiety

Study	Experimental group		Control group		Between-group significance
	Baseline	Post-intervention	Baseline	Post-intervention	
Strength outcomes					
Cornette, (2016) [24]	25.3(8.7)	24.6(7.1)	28.7(8.4)	28.6(8.8)	0.11
Dong, (2019) [25]	16.1(5.0)	20.5(4.1)*	18.6(4.1)	19.3(4.5)	0.017*
Galiano-Castillo (2016) [27]	19.8(14.0)	23(13.9)*	19.3(11.5)	19.1 (12.3)	0.006*
Rogers, (2023) [33]	55.4(25.5)	62.6(24.5)	53.3(20.8)	59.9(21.2)	0.72
Anxiety and depression outcomes					
Cornette, (2016) [24]	12.1(5.6)	9.8(4.2)	15.3(7.2)	14.3(3.5)	0.453
Rogers, (2023)^a^ [33]	7.1(3.9)	5.6(3.4)	7.0(3.9)	6.8(3.5)	0.276
Rogers, (2023)^b^ [33]	4.8(3.3)	3.0(2.6)	4.7(3.5)	4.3(3.1)	0.054

Values are expressed as mean (Standard deviation)

*statistical significance *p* < 0.05

^a^anxiety outcomes

^b^depression outcomes

### Anxiety and depression

Two studies measured anxiety and depression using Hospital Anxiety and Depression Scale and both showed no significant improvement (Table 2) [24, 33].

## Discussion

This meta-analysis suggested the effectiveness of telehealth exercise-based rehabilitation intervention in improving cardiorespiratory fitness, physical activity level, and fatigue among cancer patients when compared with conventional intervention. As the toxic cancer treatment effect on the cardio-respiratory system is increasingly recognized, the prominent improvement of cardiorespiratory fitness (1.68 ml/kg/min increment in peak VO2 or 104.54 m distance improvement in 6MWT) should be emphasized for cancer patients. In addition, improving physical activity and cardiorespiratory fitness among cancer patients without triggering/intensifying fatigue is challenging [34]. The reduction in fatigue suggests that telehealth is an ideal alternative to allow patients feasibility of physical activity at their own pace and convenience. The extensive use of telemonitoring (e.g., heart rate monitor, televideo, and accelerometer) and professional call support should be acknowledged to supervise/reassure the patients and prevent adverse events or non-adherence.

The adherence reported in the exercise interventions was high, averaging around 78.5%, corresponding with center-based cancer rehabilitation exercise interventions, where high adherence of around 92% has been reported [35]. Furthermore, increased cardiorespiratory fitness levels may correlate with higher exercise adherence [36]. Therefore, patients may be considered sufficiently motivated to exercise in their environment, despite differences in exercise prescription [37]. However, exercise prescription may differ between cancer subgroups. Our meta-analysis indicates that an effective aerobic exercise regimen typically involves sessions occurring two to five times per week, lasting between 20 to 50 min each, performed at a moderate intensity ranging from 60 to 80% of maximum heart rate, and may be supplemented with resistance exercise. However, while our analysis highlights the importance of resistance training in systematic cancer rehabilitation, determining the optimal prescription for telehealth cancer rehabilitation resistance exercises requires further investigation due to inconclusive muscle strength results. Previous studies have shown significant improvements in muscle strength with resistance training, underscoring its importance in cancer rehabilitation [38, 39].

The results of this meta-analysis demonstrated significant improvements in cardiorespiratory fitness and PA after telehealth cancer exercise rehabilitation compared to the control group but not for HRQoL and BMI change, contrary to previous center-based exercise rehabilitation demonstrating the efficacy of exercise in multiple cancer subgroups [40–42]. The failure to observe improvements in HRQoL contradicts the positive within-group changes reported by most studies. This discrepancy prompts a closer inspection of the measures employed or the specific aspects of HRQoL affected by telehealth interventions, as well as the potential influence of the control group's instructions from healthcare professionals adhering to international guidelines. Discrepancies in HRQoL may stem from nuanced measurement aspects, while BMI findings are limited by a small study pool and a proportion of patients without baseline abnormalities. The small number of studies and some proportion of patients without BMI abnormality at baseline may explain the lack of effect on BMI. It is also likely that the short duration of exercise would not be expected to necessarily alter BMI, nor would this necessarily be desirable in a group of patients with cancer unless accompanied by improvements in muscle strength. On the other hand, the importance of considering the duration and intensity of interventions in future studies is needed to stress. Finally, improved cardiorespiratory fitness and optimal PA support the clinical relevance of developing telehealth exercise-based cancer rehabilitation interventions because they reduce mortality risk and cancer burden [43–46]. A more comprehensive assessment considering psychosocial well-being and disease-related symptoms is essential for a holistic understanding of telehealth interventions in cancer rehabilitation.

In addition, longer-term maintenance is necessary to preserve the health benefits and reduced risk. Part of included study sample examined the long-term effect (six-month to two-year follow-up), and although it would be reasonable to assume that telehealth exercise would lead to longer-term improvements in clinical outcomes as has been demonstrated in chronic populations elsewhere [47–49], more robust evidence based on a methodologically rigorous design will be needed to provide satisfactory long-term evidence in survivors [50–52]. However, limited reports suggest that telehealth exercise interventions have the potential to be an effective strategy for maintaining health benefits.

Furthermore, it is important to discuss exercise safety, as many clinicians concerning exercise without direct professional supervision, as is standard with center-based rehabilitation models. Patients may be at risk of safety and adherence to an exercise prescription, leading to unsatisfactory results. In our meta-analysis, half of the studies reported adverse events, and only Rogers et al. reported a single severe exercise-related event consistent with the low rates reported in home-based exercise for cardiac population [53]. However, comparative research with supervised care in a sufficiently large study sample and inclusion of adverse event reporting in future controlled trials is needed to conclude exercise-related risk in cancer survivors.

As noted above, increased cardiovascular risk and a higher prevalence of cardiovascular risk factors have been found in survivors [54]. In focus on core cardiac preventive components [55, 56], only minor studies included cardiac output [26, 29]. Therefore, future research needs to include the systematic cardiac assessment of core prevention components that can potentially optimize cardiovascular risk, especially lipid, blood pressure, or diabetes management, through exercise-based rehabilitation [57–59]. Ultimately, exercise-based telehealth is an alternative form to delivering cancer rehabilitation exercise services through information and telecommunication technologies (PC, smartphone, internet, and videoconferencing) [8]. Based on this, the use of telehealth platforms may lead to increased attractiveness and utility in cancer exercise rehabilitation [60]. However, the acceptability and usefulness of the telemedicine approach may be limited by factors such as the validity of technological tools, technological literacy or legal clarity, and data protection [12]. Despite these telehealth challenges, the recent pandemic has dramatically promoted the use of providing digital healthcare strategies that have the potential to overcome barriers, reduce costs, and increase overall cancer exercise rehabilitation utilization [61–63].

The COVID-19 pandemic has had a significant impact on the normalization and adoption of telehealth-delivered cancer exercise interventions. The need for physical distancing and minimizing in-person interactions during the pandemic has accelerated the use of telehealth as a means of delivering healthcare services, including rehabilitation exercise interventions, to cancer patients. Telehealth allows healthcare providers to remotely monitor and guide exercise interventions, reducing the need for in-person visits and minimizing the risk of exposure to the virus. This has not only ensured the safety of cancer patients but has also provided them with the necessary support and guidance to maintain their physical activity levels during a time when access to traditional exercise facilities may be limited [64].

Ultimately, the COVID-19 pandemic has had a transformative impact on telehealth-delivered cancer exercise interventions. It has accelerated the adoption and acceptance of telehealth platforms, highlighted the importance of telehealth in ensuring continuity of care, and prompted policy changes to support its widespread use. Telehealth has become a vital tool in delivering exercise interventions to cancer patients, providing them with safe and accessible care during a time of restricted in-person interactions. The lessons learned from the pandemic will likely shape the future of cancer care, with telehealth playing an increasingly prominent role in delivering exercise interventions and supporting the overall well-being of cancer patients.

### Limitations

Since a wide range of eligible RCTs were identified to provide a broad perspective on this new area, there are limitations. Firstly, there was language bias as only evidence studies in English were included. For another, only RCT designs are limiting for pragmatic studies in clinical settings. The sample size of some studies analyzed was small, which may lead to the reliability of the results. Also, the limitation of methodologically different exercise prescriptions and heterogeneity of the measurement instruments should be mentioned, which may have affected the results. Secondly, there is heterogeneity in the definition of telehealth used in cancer exercise interventions. Pilot studies have found telehealth exercise interventions to be feasible and generally accepted among participants, but there is marked heterogeneity in specific exercise activities and telehealth modalities [18]. Thirdly, the different PA recommendations of the control groups could have affected the study findings by potentially reducing the observed differences between the control and intervention groups, impacting participant motivation levels, and introducing confounding variables related to baseline activity levels and the effectiveness of the intervention.

Finally, the meta-analysis results underscore the effectiveness of telehealth exercise-based cancer rehabilitation in improving cardiorespiratory fitness and PA. However, an important avenue for discussion revolves around determining the most effective doses needed to achieve these benefits. The study lacks detailed insights into optimal exercise duration, frequency, and intensity for meaningful outcomes. Future research should delve into these specifics to establish evidence-based guidelines, ensuring the maximum benefits are derived from telehealth interventions in cancer rehabilitation. Clarifying dosage parameters [50] will contribute to the refinement and standardization of telehealth programs, enhancing their impact on patients' well-being and overall outcomes.

## Conclusion

In summary, the results of this meta-analysis showed that telehealth exercise-based cancer rehabilitation interventions could significantly increase cardiorespiratory fitness and PA levels and reduce fatigue. However, these interventions did not significantly improve BMI, quality of life, and muscle strength. Nevertheless, these exercise interventions confirmed high adherence, which lends to further research needed to ensure development in this area. Given the limitations of this meta-analysis and the flaws in methodology rigor (unclear description in randomization and allocation and a lack of blinding), the results need to be interpreted cautiously. High-quality and robust RCTs are needed to investigate specific home-based exercise regimens in different subgroups of patients and cancer survivors.

### Supplementary Information

Supplementary Material 1.

## Data Availability

All data generated or analysed during this study are included in this published article [and its supplementary information files].

## Abbreviations

PICOS: Populations, interventions, comparisons, outcomes, and study designs
RCTs: Randomized controlled trials
SMD: Standard mean difference
MD: Mean difference
CI: Confidence interval
HRQoL: Health related quality of life
6MWT: Six-minute walk test
HR: Heart rate
PA: Physical activity
BMI: Body mass index
