# Supplementary material for: Effect of exercise-based cancer rehabilitation via telehealth: a systematic review and meta-analysis
Source: BMC Cancer. 2024 May 17;24:600. doi: 10.1186/s12885-024-12348-w (PMC11100177; doi:10.1186/s12885-024-12348-w)
Supplement: Supplementary file 1 — Supplementary Material 1. [file 12885_2024_12348_MOESM1_ESM.docx]

Supplementary Table S1. Search strategy

| Electronic databases | Search term |
| --- | --- |
| Pubmed | (Home-based exercise OR Digital health OR Telehealth OR Telerehabilitation OR Mobile health OR Remote) AND (cancer OR cancer survivors OR adjuvant therapy OR Cancer treatment OR chemotherapy OR hematology OR oncology)  Filters: Full text, Clinical Trial, Randomized Controlled Trial, from 2000 - 2023 |
| Web of Science | ((((((((TI=(Home-based exercise )) OR TI=(Digital health )) OR TI=(Telehealth )) OR TI=(Telerehabilitation )) OR TI=(Mobile health)) OR TI=(Remote )) AND TI=(cancer ))  Filters: Article, from 2000-01-01 – 2023-03-01 |

Supplementary Table S2. Adverse events and adherence

| Study | Adverse events | Adherence with exercise protocol |
| --- | --- | --- |
| Cornette (2016) | No adverse events | 88% of prescribed sessions (109% aerobic exercise, 46% resistance exercise) |
| Dong (2019) | NR | NR |
| Falz (2023) | 18 adverse events in 16 patients (11%) classified as serious adverse events; unrelated to the exercise | 74.2% performed at least 1.5 training sessions per week and 164 minutes total activity >3 MET per week across the entire study period;  56.4% interventional patients exercised more than 2 sessions per week and were active 172 minutes per week;  Reductions in adherence were mainly due to clinical factors (n=6), personal (n=2), and motivational problems (n=8). |
| Gallian-Castillo (2017) | No remarkable health problems recorded | 93.9% of prescribed sessions |
| Gehring (2018) | No serious adverse events (one pre-existing osteoarthritis-related knee pain at 24th week of intervention) | 79% of prescribed sessions (mean 2.4 sessions/week) |
| Lahart (2017) | NR | NR |
| Ligibel (2011) | NR | 30/61 participants reported daily steps for greater than 90% of days during intervention, and 9/61 reported more than 50% of days. Participants reported a mean of 153.6 min of moderate exercise per week and a 7392 steps per day. |
| McNeil (2019) | NR | 111% of prescribed PA in higher-intensity group 309% of prescribed PA in lower-intensity group |
| Pinto (2013) | NR | 65% of participants achieved 150 min/week of prescribed PA |
| Rogers (2023) | 1 exercise related serious event occurred (i.e., pelvic stress fracture);  12 unrelated serious adverse events occurred (hospitalization for TIA, thyroid cancer, infection, breast reconstruction surgery, ovarian cancer recurrence, surgery for herniated disc, breast cancer recurrence, hospitalization for knee replacement, death (details unknown), and leg fracture). | Meet recommendations (accelerometer) 71.3% intervention group vs. 57.6% control group |

NR = not reported, PA = physical activity, ADT = androgen deprivation therapy, VO_2_ = oxygen consumption, HB = home-based exercise.

Supplementary Table 3. Overview of results and methods in all studies (n=10)

| **Outcome** | **Instrument/methods and number of studies** |
| --- | --- |
| Cardiorespiratory fitness (n=10) | VO2peak testing using a modified Bruce treadmill protocol, n=3, [26,30,33]  6-minute walk test, n=3, [25,28,31]  VO2max or VO2peak assessed using a cycle ergometer, n=2, [27,29]  Submaximal Balke treadmill test, n=1, [32]  Submaximal treadmill test using the Naughton protocol n=1, [34] |
| Health-related Quality of Life (n=7) | EORTC QLRC30, n=4, [25,27,28,31]  Medical Outcomes Study 36-item short-form survey, n=3, [26,33,34] |
| Physical activity (n=7) | IPAQ, n=3, [29,30]  7-Day Physical Activity Recall, n=2, [31,33]  MVPA assessed with the ActiGraph® GT3X+ accelerometer, n=2, [32,34]  Activity per week, n=1, [27] |
| Fatigue (n=5)^a^ | Multidimensional Fatigue Inventory, n=1, [25]  Piper Fatigue Scale, n=1, [28]  The Functional Assessment of Chronic Illness Therapy – Fatigue Scale, n=1, [31]  FACT-F, n=1, [33]  Fatigue Symptom Inventory, n=1, [34] |
| Body mass index (n=5) | n=5, [25,27,29,32,34] |
| Strength (n=4) | Handgrip dynamometer, n=1 [28]  Leg dynamometer, n=1, [34]  Isometric bench n=1, [25]  Arm lifting test n=1, [26] |
| Anxiety and depression (n=2) | Hospital Anxiety and Depression Scale, n=2, [25,34] |

EORTC QLRC30, EORTC Core Quality of Life questionnaire; FACT-F, Functional Assessment of Cancer Therapy: Fatigue; IPAQ, International Physical Activity Questionnaire; MVPA, Moderate to Vigorous Physical Activity; VO2peak, Peak oxygen uptake; ^a^, Higher scores indicate a higher level of fatigue

Supplementary Figure 1 Subgroup and sensitivity analysis


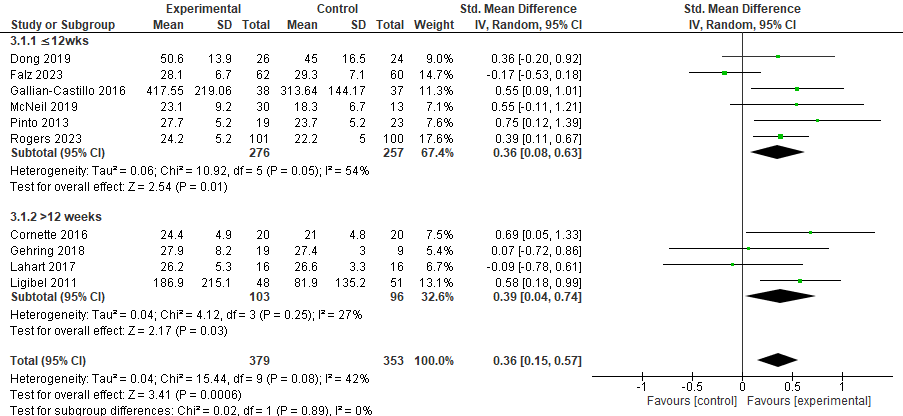


Figure 1a. Subgroup analysis: effect of telehealth cancer exercise on cardiorespiratory fitness (duration)


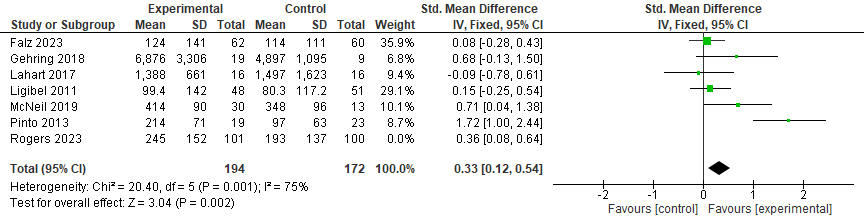


Figure 1b, sensitivity analysis: effect of telehealth cancer exercise on physical activity (removing studies that provide supervised exercise training to the control group)


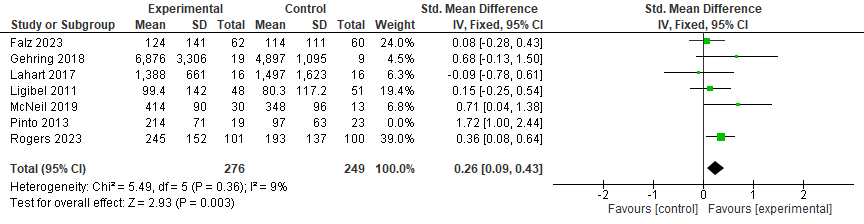


Figure 1c: Sensitivity analysis: effect of telehealth cancer exercise on physical activity (removing one study with high effect size)
